# Daptomycin-Loaded Nano-Drug Delivery System Based on Biomimetic Cell Membrane Coating Technology: Preparation, Characterization, and Evaluation

**DOI:** 10.3390/ph18081169

**Published:** 2025-08-06

**Authors:** Yuqin Zhou, Shihan Du, Kailun He, Beilei Zhou, Zixuan Chen, Cheng Zheng, Minghao Zhou, Jue Li, Yue Chen, Hu Zhang, Hong Yuan, Yinghong Li, Yan Chen, Fuqiang Hu

**Affiliations:** 1 School of Pharmacy, China Pharmaceutical University, No. 639, Longmian Avenue, Nanjing 211198, China; 2 NMPA Key Laboratory for Testing and Warning of Pharmaceutical Microbiology, Zhejiang Key Laboratory of Biopharmaceutical Contact Materials, Zhejiang Institute for Food and Drug Control, No. 325 Pingle Street, Binjiang District, Hangzhou 310052, China; 3 School of Pharmaceutical Sciences, Hangzhou Medical College, Hangzhou 311399, China; 4 Institute of Agro-Product Safety and Nutrition, Zhejiang Academy of Agricultural Sciences, No. 198 Shiqiao Road, Hangzhou 310021, China; 5 College of Pharmaceutical Science, Zhejiang University, No. 866 Yuhangtang Road, Hangzhou 310058, China; 6 Department of Infectious Diseases, Sir Run Run Shaw Hospital, Zhejiang University School of Medicine, Hangzhou 310016, China

**Keywords:** daptomycin, M1-type macrophage membrane, Staphylococcus aureus, drug-resistance, zebrafish

## Abstract

**Background/Objective:** Staphylococcus aureus (*S. aureus*) is a clinically significant pathogenic bacterium. Daptomycin (DAP) is a cyclic lipopeptide antibiotic used to treat infections caused by multidrug-resistant Gram-positive bacteria, including *S. aureus*. However, DAP currently faces clinical limitations due to its short half-life, toxic side effects, and increasingly severe drug resistance issues. This study aimed to develop a biomimetic nano-drug delivery system to enhance targeting ability, prolong blood circulation, and mitigate resistance of DAP. **Methods:** DAP-loaded chitosan nanocomposite particles (DAP-CS) were prepared by electrostatic self-assembly. Macrophage membrane vesicles (MM) were prepared by fusion of M1-type macrophage membranes with 1,2-dimyristoyl-sn-glycero-3-phosphocholine (DMPC). A biomimetic nano-drug delivery system (DAP-CS@MM) was constructed by the coextrusion process of DAP-CS and MM. Key physicochemical parameters, including particle diameter, zeta potential, encapsulation efficiency, and membrane protein retention, were systematically characterized. In vitro immune escape studies and in vivo zebrafish infection models were employed to assess the ability of immune escape and antibacterial performance, respectively. **Results:** The particle size of DAP-CS@MM was 110.9 ± 13.72 nm, with zeta potential +11.90 ± 1.90 mV, and encapsulation efficiency 70.43 ± 1.29%. DAP-CS@MM retained macrophage membrane proteins, including functional TLR2 receptors. In vitro immune escape assays, DAP-CS@MM demonstrated significantly enhanced immune escape compared with DAP-CS (*p* < 0.05). In the zebrafish infection model, DAP-CS@MM showed superior antibacterial efficacy over both DAP and DAP-CS (*p* < 0.05). **Conclusions:** The DAP-CS@MM biomimetic nano-drug delivery system exhibits excellent immune evasion and antibacterial performance, offering a novel strategy to overcome the clinical limitations of DAP.

## 1. Introduction

Staphylococcus aureus (*S. aureus*) is one of the most prevalent community- and hospital-acquired pathogens and causes a wide range of local and systemic infections. As a first-line antibiotic against *S. aureus* infections, daptomycin (DAP) exerts rapid bactericidal effects through the disruption of bacterial membrane potential and inhibition of RNA/DNA synthesis [1]. DAP is a concentration-dependent antibiotic [2]. To exert its antibacterial effect, it needs to maintain a certain effective bactericidal concentration in vivo. However, the half-life of the common DAP formulation is short. Repeated use of DAP inevitably produces toxicity to normal tissues and organs, which will lead to the emergence of drug-resistant bacteria, thereby reducing the efficacy. In recent years, daptomycin-resistant *S. aureus* has emerged in clinical practice [3,4,5,6], and its resistance mechanism mainly involves the adaptive changes in bacterial cell membrane structure and function. The resistance of these strains is usually mediated by mutations in the MprF gene [7]. Therefore, it is urgent to develop a new formulation with better efficacy, lower toxicity, and less antibiotic resistance.

In recent years, nano-drug delivery systems have attracted much attention and are increasingly used in the field of medicine [8,9,10,11]. However, the clinical application of nano-drug delivery systems still faces challenges such as the formation of protein crowns in vivo [12], the rapid clearance mechanism of the immune system [13], and the lack of targeting for deep infection [14]. Hence, researchers have turned their attention to biomimetic nano-drug delivery systems, hoping to improve the biocompatibility and targeting capacity with the aid of endogenous carriers. To avoid the interaction between living cells and nano-drug carriers, researchers have extracted and separated the cell membrane of living cells and integrated it into the surface of the nano-drug carriers. Biochemical nano-drug delivery system has the advantages of both endogenous carrier systems and nano-drug carriers, which have great potential in the field of drug delivery research [15,16].

Macrophage membrane biomimetic nano-drug delivery system has achieved certain results and shows unique advantages [17,18,19,20,21]. Geng et al. designed a macrophage membrane biomimetic nano-delivery system (MA@RT-HMSNs), which can effectively cross the blood-brain barrier and accurately deliver TC-DAPK6 to the inflammatory site of epileptic focus, significantly enhancing the bioavailability of TC-DAPK6 [22]. Li et al. constructed macrophage membrane (MM)-wrapped photosensitizer-loaded collagen nanoparticles (Ce6/Col/MM), which can be used to treat wound infection caused by drug-resistant bacteria and promote wound healing [23]. The breakthrough advantages of using macrophage membrane-coated core-shell nano systems are reflected in enhanced immune escape, precise targeting of lesions, and intelligent drug delivery. Thus, a macrophage membrane biomimetic nano-drug delivery system can reduce immunogenicity, prolong systemic circulation, and enhance immune evasion and targeting capabilities.

In this study, we designed a biomimetic nano-drug delivery system (DAP-CS@MM) with DAP-loaded chitosan nanocomposite particles (DAP-CS) as the core and macrophage membrane vesicles (MM) as the outer shell. MM was composed of 1,2-dimyristoyl-sn-glycero-3-phosphocholine (DMPC) and M1-type macrophage membrane. TLR2 receptors on M1-type macrophage membranes can mediate specific binding to staphylococcal lipoproteins [24], allowing DAP-CS@MM to inflammatory sites. In this study, the immune escape ability of DAP-CS@MM was studied in vitro. Also, the antibacterial efficacy of DAP-CS@MM in zebrafish infection models induced by drug-resistant *S. aureus* was evaluated.

## 2. Results and Discussion

### 2.1. Characterization of M1-Type Macrophage Membrane

**Figure 1:**
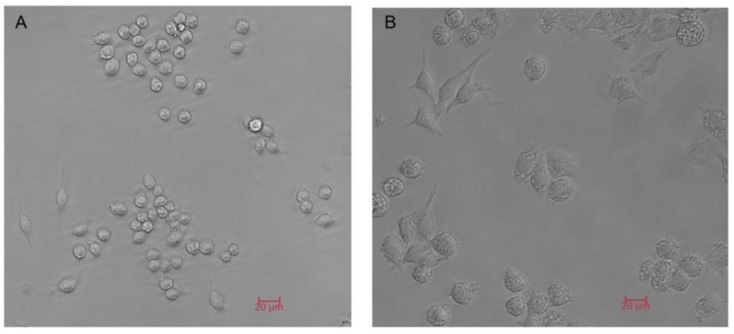
Morphological characters of Raw264.7 under the inverted phase contrast microscope (×200). (**A**) The morphology of macrophages without stimulation. (**B**) The morphology of macrophages with the stimulation of LPS.

The morphology of macrophages was observed by an inverted phase contrast microscope (DM IL, Leica, Wetzlar, Germany). Unstimulated macrophages displayed obvious clustered growth patterns with round or oval shapes (Figure 1A). After 12 h of Lipopolysaccharide (LPS) stimulation, the pseudopods of macrophages in the peripheral appeared more and longer, and the cell bodies were stellate or irregular in shape (Figure 1B). These morphological changes preliminarily indicate that macrophages can polarize to M1-type macrophages with the stimulation of LPS.

**Figure 2:**
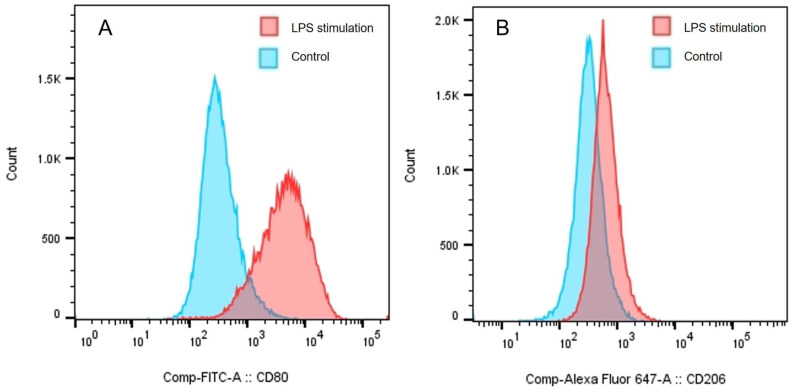
The specific protein expression of CD80 (**A**) and CD206 (**B**) in macrophages.

M1-type and M2-type macrophages exhibited significant CD80 expression. However, M2-type macrophages specifically expressed CD206, which was absent in M1-type macrophages. Thus, the polarization of macrophages to M1-type can be identified by detecting the expression of CD80 and CD206 [25,26,27,28,29]. With the stimulation of LPS, the expression of CD80 on the surface of macrophages was increased significantly (Figure 2A), and CD206 was expressed negatively (Figure 2B). These results indicated the successful polarization of M1-type macrophages.

### 2.2. Particle Size, Zeta Potential, and Encapsulation Efficiency

DAP-CS were successfully prepared by electrostatic self-assembly, driven by the interaction between positively charged CS and negatively charged DAP at pH 5. As shown in Table 1, DAP-CS exhibited a small size (90.93 ± 5.38 nm) and high positive zeta potential (27.48 ± 0.53 mV), indicating stable colloidal dispersion due to strong electrostatic repulsion. The encapsulation efficiency (76.09 ± 0.42%) was attributed to electrostatic interaction between DAP and CS under optimized preparation conditions. Upon coating with MM (forming DAP-CS@MM), the particle size increased by ~20 nm (110.9 ± 13.72 nm), confirming successful MM deposition on the nanoparticle surface. The reduced zeta potential (11.90 ± 1.90 mV) suggested partial charge shielding by the MM, which carried negative charges. The decreased encapsulation efficiency (70.43 ± 1.29%) could be due to minor drug leakage during the coextrusion process of DAP-CS and MM. In summary, both DAP-CS and DAP-CS@MM nanoparticles were successfully prepared, exhibiting small particle sizes, uniform dispersion, appropriate surface charge, and high encapsulation efficiency.

### 2.3. Characterization of Membrane Proteins

**Figure 3:**
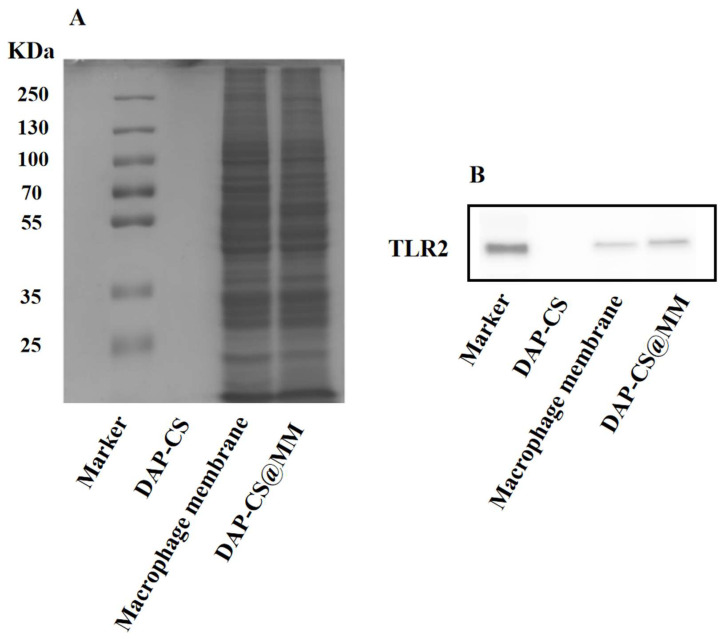
Membrane protein characterization of DAP-CS, macrophage membrane, and DAP-CS@MM. (**A**) Protein profiles of DAP-CS, macrophage membrane, and DAP-CS@MM analyzed using SDS-PAGE. (**B**) Expression of TLR2 in samples measured using western blotting.

We anticipated that membrane-associated protein content would be translocated onto the biomimetic nanoparticles. Thus, the protein composition of DAP-CS@MM was analyzed using SDS-PAGE to validate the biomarker profiles (Figure 3A). The macrophage membrane was included in parallel for comparison. Obviously, DAP-CS@MM inherited a very similar proteinogram from macrophages, thereby enhancing adhesion to the inflammation location. TLR2 receptors on M1-type macrophage membranes can mediate specific binding to staphylococcal lipoproteins, allowing DAP-CS@MM to reach inflammatory sites. We further examined the TLR2 of DAP-CS@MM using a Western blot. Using specific antibodies against TLR2, the proteins were identified in both the macrophage membrane and DAP-CS@MM, whereas no band was observed for DAP-CS (Figure 3B). These results confirmed that DAP-CS and macrophage membrane were assembled by physical fusion, and predicted that DAP-CS@MM will possess similar inflammatory chemotaxis as M1-type macrophages.

### 2.4. Immune Escape Assay In Vitro

**Figure 4:**
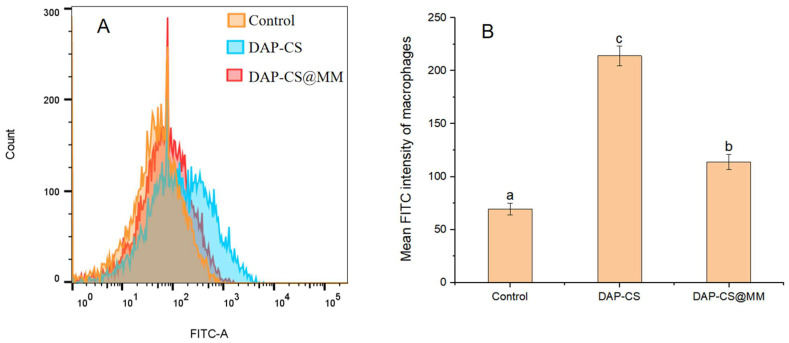
Analysis of macrophage uptake for different formulations by flow cytometry (*n* = 3). (**A**) Flow cytometry analysis of FITC in macrophages after incubation with DAP-CS-FITC or DAP-CS@MM-FITC. (**B**) Quantitative analysis of mean FITC intensity of macrophages. (Data are presented as the means ± SD. Groups sharing the same letter are not significantly different (*p* > 0.05). Groups labeled with different letters (a–c) are statistically significant (*p* < 0.05)).

We evaluated the cellular uptake of DAP-CS and DAP-CS@MM in macrophages. After an incubation of DAP-CS-FITC and DAP-CS@MM-FITC with RAW264.7 at 37 °C for 4 h, the intracellular FITC intensity was quantified using flow cytometry. Compared with DAP-CS, we detected decreased FITC intensities in DAP-CS@MM (Figure 4A). The mean FITC intensity of the macrophages quantified by flow cytometry was 69.47 ± 5.77 (control), 214 ± 9.17 (DAP-CS), and 114 ± 7.00 (DAP-CS@MM). Compared with DAP-CS, DAP-CS@MM showed a notably lower FITC intensity of macrophages (Figure 4B) (*p* < 0.05). These results indicated that DAP-CS@MM had the function of immune escape. The stealth properties of DAP-CS@MM nanoparticles are mediated by preserved immunomodulatory proteins in the macrophage membrane coating that confer immune evasion capabilities.

### 2.5. Evaluation of Antibacterial Efficacy In Vivo

#### 2.5.1. Determination of Maximum Tolerated Dose

As shown in Table 2, no zebrafish mortality was observed after injecting DAP at a dose range of 125 to 1000 ng/larva. Mortality rate increased to around 17% (5/30) when the dose was 2000 ng/larva. Compared with the model control group, the behaviors of zebrafish were normal at 250 ng/larva and 125 ng/larva, while they became abnormal at 500 ng/larva and 1000 ng/larva. Based on the above results, the maximum tolerated dose (MTD) of DAP was determined to be 250 ng/larva, which could ensure the antibacterial effect without causing obvious toxic effects. No mortality or abnormal behaviors were observed in both the normal control group and model control group, indicating that the experimental system was stable and reliable.

#### 2.5.2. Assessment of Antibacterial Efficacy

**Figure 5:**
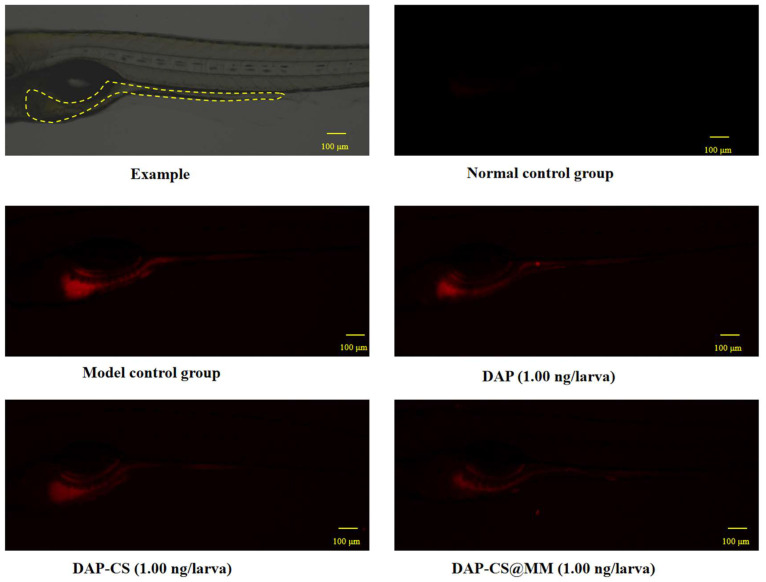
Representative images of bacterial fluorescence intensity in zebrafish following experimental treatment.

The antibacterial efficacy was evaluated through integrated analysis of quantitative fluorescence intensity measurements (Table 3) and qualitative fluorescence imaging (Figure 5). In Figure 5, fluorescence imaging revealed enhanced red fluorescence signal in the model control group compared to the normal control, confirming successful infection establishment. Notably, the DAP-CS@MM treatment group exhibited the weakest fluorescence intensity among all experimental groups, demonstrating superior antibacterial activity. Quantitative analysis corroborated these observations. In Table 3, the bacterial fluorescence intensity in the model control group (864,890 ± 39,470) demonstrated a highly significant increase (*p* < 0.05) compared with the normal control group (45,925 ± 3577), confirming successful establishment of the infection model. The fluorescence intensity of DAP (766,996 ± 16,054), DAP-CS (754,545 ± 32,188), and DAP-CS@MM (660,482 ± 29,038) groups was significantly lower compared with the model control group, indicating that these groups all had antibacterial activity. Moreover, the antibacterial efficacy of DAP-CS@MM was superior to DAP and DAP-CS (*p* < 0.05).

LAC-D8 was laboratory-derived daptomycin-resistant *S. aureus* isolates, which exhibited reduced susceptibility to antibiotics. These results indicated DAP-CS@MM could offer a promising strategy against daptomycin-resistant *S. aureus* infections.

This study developed a biomimetic nano-drug delivery system (DAP-CS@MM) to overcome the clinical limitations of daptomycin (DAP) by integrating macrophage membrane (MM) coating with DAP-CS. The MM coating enabled immune evasion by maintaining immunomodulatory proteins, resulting in significantly lower macrophage uptake than DAP-CS (*p* < 0.05). Additionally, TLR2-mediated pathogen recognition enhanced bacterial targeting, while the nanosized structure promoted accumulation at infection sites. In a zebrafish *S. aureus* infection model, DAP-CS@MM demonstrated superior antibacterial efficacy over free DAP and DAP-CS (*p* < 0.05), attributed to prolonged circulation, targeted delivery, and potential synergy between DAP and MM-derived antimicrobial components.

## 3. Materials and Methods

### 3.1. Materials

#### 3.1.1. Chemicals and Reagents

DAP (CAS: 103060-53-3) was purchased from the National Institutes for Food and Drug Control (Beijing, China). CS with molecular weight 20 kDa and deacetylation degree 95% was obtained from Yuhuan Marine Bio-Chemical Co., Ltd. (Yuhuan, China). DMPC was sourced from Aladdin Biochemical Technology Co., Ltd. (Shanghai, China). LPS, radio immunoprecipitation assay lysis buffer (RIPA), protease inhibitors, membrane and cytosol protein extraction kit, and SDS-PAGE kits were acquired from Beyotime Biotechnology (Shanghai, China). Dulbecco’s modified Eagle’s medium (DMEM), fetal bovine serum (FBS), and phosphate-buffered saline (PBS) were obtained from Gibco, Thermo Fisher Scientific (Grand Island, NY, USA). FITC was bought from Acros Organic (New York, NY, USA). PE Rat Anti-Mouse CD86 and FITC Hamster Anti-Mouse CD80 were obtained from BD Biosciences (San Jose, CA, USA). Rabbit anti-mouse TLR2 protein was obtained from Abcam company (Cambridge, MA, UK). Goat Anti-Rabbit IgG (H+L) HRP was sourced from Bioworld Technology, Inc. (Dublin, OH, USA). Vybrant^TM^ Dil Cell Labeling Solution (CM-DiI) was procured from Thermo Fisher Scientific Co., Ltd. (Shanghai, China). 0.9% Sodium Chloride Injection was purchased from Hunan Kelun Pharmaceutical Co., Ltd. (Yueyang, China). Trypticase soy broth was acquired from Qingdao Hi-tech Industrial Park Hope Bio-technology Co., Ltd. (Qingdao, China).

#### 3.1.2. Biological Materials

RAW264.7 cells were purchased from Zhejiang Meisen Cell Technology Co., Ltd. (Panan, China). The cells were cultured in DMEM with 10% FBS at 37 °C in a 5% CO_2_ incubator (HERAcell 150i, Thermo Scientific, Waltham, MA, USA) with >95% humidity.

LAC-D8 was laboratory-derived daptomycin-resistant *S. aureus* isolates, which were obtained from Sir Run Run Shaw Hospital (Hangzhou, China) [30]. Strains were grown in tryptic soy broth.

Wild-type AB strain zebrafish embryos (6 h postfertilization) were supplied by Hangzhou Hunter Biotechnology Co., Ltd. (Hangzhou, China). Zebrafish were maintained in automated systems under controlled conditions: 28 °C, pH 6.5~8.5, salinity 0.02%, conductivity 450~550 µs·cm^−1^.

### 3.2. M1-Type Macrophage Membranes

#### 3.2.1. Polarization and Characterization

RAW264.7 cells were stimulated with l μg·mL^−1^ LPS for 12 h to obtain M1-type macrophages [31]. Following collection, cells were washed twice with PBS and resuspended in 100 μL flow cytometry staining buffer. For surface marker analysis, cells were stained with 5 μL PE Rat Anti-Mouse CD86 and 5 μL FITC Hamster Anti-Mouse CD80, followed by 30 min incubation at 4 °C protected from light. Then the cells were washed twice with PBS, resuspended in 500 μL of PBS, and observed M1-type macrophages polarization on a flow cytometer (BD FACSVERSE, Becton Dickinson, Franklin Lakes, NJ, USA). The macrophages without LPS polarization were set as the control group.

#### 3.2.2. Extraction of M1-Type Macrophage Membranes

In this study, repeated freeze–thaw cycles were employed to lyse cells, followed by removal of soluble protein fractions via density gradient centrifugation to yield purified membrane preparations. The specific procedure was as follows: the polarized M1-type macrophages were resuspended in PBS, frozen at −80 °C for 10 min, thawed in a water bath at 37 °C, and repeatedly frozen–thawed 3 times. The lysates were first centrifuged (5430R, Eppendorf, Hamburg, Germany) at 3000×* g* for 5 min at 4 °C to remove cellular debris. Then the supernatant was collected and further centrifuged at 10,000×* g* for 5 min at 4 °C to obtain the cell membrane precipitate. The obtained cell membrane precipitate was resuspended in purified water to achieve a final membrane protein concentration of 0.25 mg·mL^−1^ and stored at −80 °C for later use.

### 3.3. Preparation of Biomimetic Nano-Drug Delivery System

#### 3.3.1. Preparation of DAP-CS

The selection of CS was based on its cationic nature, enabling electrostatic interaction with DAP at pH 5. DAP-CS was prepared at three mass ratios (1:0.5, 1:5, and 1:10 *w*/*w*). Systematic characterization revealed the 1:0.5 ratio exhibited optimal physicochemical properties (Table S1). For the selected formulation, 5 mg chitosan was precisely weighed and dissolved in 5 mL of distilled water. Then the pH was adjusted to 5 using acetic acid. A 10 mg amount of DAP was accurately weighed and dissolved in 10 mL of distilled water. The DAP solution was then mixed with chitosan solution under magnetic stirring (C-MAG HS 7, IKA, Staufen, Germany) for 10 min. The resulting DAP-CS nanocomposite suspension was diluted with distilled water to a final DAP concentration of 100 μg·mL^−1^ for subsequent use.

#### 3.3.2. Phospholipid Materials

The selection of DMPC was based on its properties that promote membrane fusion through its phase transition behavior near physiological temperatures and enhance membrane fluidity for improved structural stability. DMPC suspension was prepared by thin-film dispersion method as follows. A 10 mg amount of DMPC was accurately weighed into a 500 mL round-bottom flask, dissolved in 10 mL chloroform, and formed into a thin film using a rotary evaporator (RV10, IKA, Staufen, Germany). The evaporation procedure was conducted at 37 °C under reduced pressure (206 mbar) with rotation at 70 rpm for 30 min to ensure complete solvent removal. Subsequently, 10 mL distilled water was added for hydration (37 °C, 70 rpm, 40 min), yielding 1 mg·mL^−1^ DMPC suspension for subsequent use.

#### 3.3.3. Preparation of DAP-CS@MM

To prepare biomimetic DAP-CS@MM, 2 mL of the membrane solution (0.25 mg·mL^−1^ membrane protein, as described in Section 3.2.2) was mixed with 0.5 mL of 1 mg·mL^−1^ DMPC suspension (as detailed in Section 3.3.2). The mixture was sonicated in an ice-water bath for 10 min and extruded through a 200 nm polycarbonate membrane using a liposome extruder (Jungao Biotechnological Co., Ltd., Guangzhou, China) for 10 cycles to obtain the membrane vesicles (MM). DAP-CS@MM was prepared at varying volume ratios (1:0.5:0.5, 1:0.5:1, and 1:0.5:2 *v*/*v*/*v*) to evaluate the effect of membrane incorporation. Based on physicochemical characterization (Table S2), the 1:0.5:0.5 ratio was selected as optimal for subsequent studies. The final DAP-CS@MM was prepared by combining 0.5 mL of MM vesicles with 9.5 mL of 100 μg·mL^−1^ DAP-CS nanocomposite solution (prepared in Section 3.3.1), followed by identical coextrusion process (10 passes through 200 nm polycarbonate membranes). DAP-CS@MM was prepared just before use. The percentage composition of each formulation component is presented in Table S3.

### 3.4. Characterization of DAP-CS@MM

#### 3.4.1. Particle Size and Zeta Potential

The particle size, zeta potential, and PDI of both DAP-CS and DAP-CS@MM were measured by dynamic light scattering (DLS) using a Zetasizer analyzer (Zetasizer pro, Malvern Panalytical, Malvern, UK). Samples were diluted to the appropriate concentrations, and 1mL sample was taken for detection. Triplicate measurements were performed for each sample, with results reported as mean ± standard deviation.

#### 3.4.2. Encapsulation Efficiency

Separation of free drug and drug-loaded nanoparticles was achieved by ultracentrifugation, followed by encapsulation efficiency (EE) determination via HPLC method. A 10 mL amount of the sample solution was transferred to pre-cooled ultracentrifuge tubes and centrifuged at 20,000×* g* for 40 min at 4 °C. The supernatant containing free DAP was quantified using an HPLC system (LC-20AT, Shimadzu, Kyoto, Japan) equipped with a CLC-C8(M) reversed-phase column (150 mm × 4.6 mm, 5 μm; Shimadzu) and a diode array detector (DAD). The mobile phase consisted of ammonium dihydrogen phosphate buffer (4.5 g·L^−1^, pH 3.25 adjusted with phosphoric acid) and acetonitrile (60:40, *v*/*v*) at a flow rate of 1.0 mL·min^−1^. Column temperature was maintained at 25 °C with detection at 214 nm. Injection volume was 20 μL. Encapsulation efficiency was calculated as follows.

EE (%) = (C_2_ − C_1_)/C_2_ × 100%

where C_1_ represents free DAP content and C_2_ denotes the total DAP content.

#### 3.4.3. Membrane Protein Characterization

To verify retention of M1-type macrophage membrane protein composition and specific functional proteins in DAP-CS@MM, membrane proteins were initially isolated from macrophage membrane, DAP-CS, and DAP-CS@MM using a membrane and cytosol protein extraction kit. The protein profiles of macrophage membrane, DAP-CS, and DAP-CS@MM were determined by SDS-PAGE (PowerPac Universal &MP4&Mini Sub-cell, Bio-Rad, Hercules, CA, USA), and the expression of TLR2 on macrophage membrane, DAP-CS, and DAP-CS@MM was detected by Western blotting [32,33,34,35].

### 3.5. In Vitro Immune Escape Assay

DAP-CS nanocomposite solution (10 mL, 100 μg·mL^−1^) was mixed with 100 μL of FITC solution (100 μg·mL^−1^) and incubated at 37 °C for 4 h in the dark. Free FITC was removed through ultrafiltration to yield FITC-labeled DAP-CS (DAP-CS-FITC). Then, 9.5 mL of DAP-CS-FITC was combined with 0.5 mL of MM (prepared as in Section 3.3.3) and co-extruded through a 200 nm polycarbonate membrane using a liposome extruder (10 times) to generate fluorescently labeled biomimetic nanoparticles (DAP-CS@MM-FITC).

DAP-CS-FITC and DAP-CS@MM-FITC nanoparticle formulations were diluted to equivalent FITC concentrations and incubated with RAW264.7 cells. After 12 h incubation, cells were washed with PBS and resuspended for flow cytometric analysis. Fluorescence-free DAP-CS nanoparticles served as the blank control group.

### 3.6. Evaluation of Antibacterial Efficacy

#### 3.6.1. Maximum Tolerated Dose

Thirty wild-type AB strain zebrafish at 3 days post-fertilization (3dpf) were randomly selected and distributed into 6-well plates (Bioland Biotechnology Co., Ltd., Huzhou, China) in 3 mL fresh fish water. Experimental groups were injected with LAC-D8 into the yolk sac to establish the zebrafish bacterial infection models. After 24 h incubation at 28 °C, DAP was administered via intestinal injection at doses of 0, 125, 250, 500, 1000, and 2000 ng/larva. Normal control group was maintained under same conditions without any treatment. Following an additional 24 h incubation at 28 °C, the MTD was determined by inspection of infected zebrafish.

#### 3.6.2. Antibacterial Efficacy

LAC-D8 strain labeled with red-fluorescent CM-DiI (LAC-D8@CM-DiI) was prepared as previously described [36]. Wild-type AB strain zebrafish (3 dpf) were randomly allocated into 6-well plates in 3 mL fresh fish water. Experimental groups were micro-injected with LAC-D8@CM-DiI into the yolk sac to establish LAC-D8@CM-DiI infected models. After 24 h incubation at 28 °C, DAP, DAP-CS, and DAP-CS@MM with total DAP concentration of 1.0 ng/larva were micro-injected into intestine separately. Normal zebrafish model without LAC-D8@CM-DiI infection and any DAP treatment was used as normal control group. Zebrafish model with LAC-D8@CM-DiI infection while without any DAP treatment was used as model control group. Following an additional 24 h incubation at 28 °C, 10 zebrafish per group were randomly selected and imaged under fluorescence microscopy. Bacterial fluorescence intensity was quantified using NIS-Elements D3.20 software. Antibacterial efficacy was evaluated based on fluorescence intensity.

### 3.7. Statistical Analyses

All data were analyzed using SPSS 26.0 software. Data are expressed as mean ± standard deviation (SD). Data from antibacterial efficacy and immune escape assays were analyzed by one-way ANOVA followed by Tukey’s post hoc test for all multiple comparisons (α = 0.05). *p* < 0.05 was considered statistically significant. In Figures and Tables, significant differences are indicated by lowercase letters (a, b, c, etc.). Groups sharing the same letter are not significantly different (*p* > 0.05). Groups labeled with different letters are statistically significant (*p* < 0.05).

## 4. Conclusions

This study successfully developed a biomimetic nano-drug delivery system (DAP-CS@MM) by the coextrusion process of DAP-CS and MM. DAP-CS@MM exhibited optimal physicochemical characteristics with a uniform size distribution (110.9 ± 13.72 nm) and positive surface charge +(11.90 ± 1.90) mV, demonstrating excellent drug encapsulation efficiency (70.43 ± 1.29%) while preserving critical macrophage membrane proteins, including functional TLR2 receptors. The retention of these biological recognition elements enabled targeted delivery to S. aureus through pathogen-specific molecular interactions, as evidenced by superior antibacterial efficacy in zebrafish infection models where DAP-CS@MM significantly outperformed both free DAP and uncoated DAP-CS (*p* < 0.05). Moreover, the macrophage membrane coating conferred immune evasion capabilities, allowing prolonged systemic circulation as confirmed through in vitro immune escape assays (*p* < 0.05). Overall, this biomimetic nano-drug delivery system (DAP-CS@MM) has great potential for clinical treatment of drug-resistant bacterial infections.

## Figures and Tables

**Table 1:** Size and zeta potential of nanoparticles (mean ± SD, *n* = 3).

Sample	Particle Size (nm)	PDI	Zeta Potential (mV)	Entrapment Efficiency (%)
DAP-CS	90.93 ± 5.38	0.14 ± 0.06	27.48 ± 0.53	76.09 ± 0.42
DAP-CS@MM	110.9 ± 13.72	0.19 ± 0.03	11.90 ± 1.90	70.43 ± 1.29

**Table 2:** Determination of MTD (*n* = 30).

Group	Dose (ng/Larva)	Mortality (%)	Behaviors
Normal Control Group	-	0	normal
Model Control Group	0	0	normal
DAP	125	0	normal
	250	0	normal
	500	0	abnormal *
	1000	0	abnormal *
	2000	17	-

* Abnormal: exhibiting toxicity phenotypes such as body curvature, reduced locomotor activity, swim bladder disappearance, etc.

**Table 3:** Experimental results of antibacterial efficacy evaluation of samples (*n* = 10, mean ± SD).

Group	Concentration (ng/larva)	Fluorescence Intensity (Pixels)
Normal Control Group	-	45,925 ± 3577 a
Model Control Group	-	864,890 ± 39,470 d
DAP	1.00	766,996 ± 16,054 c
DAP-CS	1.00	754,545 ± 32,188 c
DAP-CS@MM	1.00	660,482 ± 29,038 b

Groups sharing the same letter are not significantly different (*p* > 0.05). Groups labeled with different letters (a, b, c, d) are statistically significant (*p* < 0.05).

## Data Availability

The data presented in this study are available in this article.

## Supplementary Materials

The following supporting information can be downloaded at: https://www.mdpi.com/article/10.3390/ph18081169/s1. Table S1: Size and zeta potential of DAP-CS at different mass ratios; Table S2: Physicochemical properties of DAP-CS@MM at different formulation volume ratios. Table S3: Composition of prepared formulations (% *w*/*v*).
