# Supplementary material for: Daptomycin-Loaded Nano-Drug Delivery System Based on Biomimetic Cell Membrane Coating Technology: Preparation, Characterization, and Evaluation
Source: Pharmaceuticals (Basel). 2025 Aug 6;18(8):1169. doi: 10.3390/ph18081169 (PMC12389226; doi:10.3390/ph18081169)
Supplement: Supplementary file 1 [file pharmaceuticals-18-01169-s001.zip › pharmaceuticals-3759575-supplementary.pdf]

Table S1. Size and zeta potential of DAP-CS at different mass ratios (mean ± SD, n=3). In blue is the selected formulation composition for the current investigation.

DAP:CS (w/w)	Size (nm)	PDI	Zeta Potential (mV)
**1:0.5**	**90.93 ± 5.38**	**0.14 ± 0.06**	**27.48 ± 0.53**
1:5	117.90± 4.21	0.17±0.10	25.68±0.47
1:10	216.30± 4.05	0.27±0.21	24.98±0.41

Table S2. Physicochemical properties of DAP-CS@MM at different formulation volume ratios (mean ± SD, n=3). In blue is the selected formulation composition for the current investigation.

DAP:CS:MM (v/v/v)	Size (nm)	PDI	Zeta (mV)
**1:0.5:0.5**	**110.9 ± 13.72**	**0.19 ± 0.03**	**11.90 ± 1.90**
1:0.5:1	338.40 ± 15.70	0.42 ±0.23	6.68 ±1.34
1:0.5:2	367.70 ± 14.89	0.35 ±0.16	4.44 ±1.79

Table S3. Composition of prepared formulations (% w/v)

Formulation	CS	Dap	DMPC	Membrane Protein
DAP-CS	0.005%	0.01%	-	-
DMPC suspension	-	-	0.1%	-
MM vesicles	-	-	0.02%	0.02%
DAP-CS@MM	0.005%	0.01%	0.001%	0.001%

"-" indicates the absence of the specified component in the formulation.
